# Analysis of Antibiotic Exposure and Development of Acute Graft-vs-Host Disease Following Allogeneic Hematopoietic Cell Transplantation

**DOI:** 10.1001/jamanetworkopen.2023.17188

**Published:** 2023-06-07

**Authors:** Armin Rashidi, Fei Gao, David N. Fredricks, Steven A. Pergam, Marco Mielcarek, Filippo Milano, Brenda M. Sandmaier, Stephanie J. Lee

**Affiliations:** 1 Fred Hutchinson Cancer Center, Seattle, Washington; 2 Biostatistics, Bioinformatics and Epidemiology Program, Vaccine and Infectious Disease Division, Fred Hutchinson Cancer Center, Seattle, Washington; 3 Division of Allergy and Infectious Diseases, Department of Medicine, University of Washington, Seattle; 4 Division of Medical Oncology, Department of Medicine, University of Washington,

## Abstract

**Importance:**

Certain antibiotic exposures have been associated with increased rates of acute graft-vs-host disease (aGVHD) after allogeneic hematopoietic cell transplantation (allo-HCT). Since antibiotic exposure can both affect and be affected by infections, analyzing time-dependent exposure in the presence of multiple potential confounders, including prior antibiotic exposures, poses specific analytical challenges, necessitating both a large sample size and unique approaches.

**Objective:**

To identify antibiotics and antibiotic exposure timeframes associated with subsequent aGVHD.

**Design, Setting, and Participants:**

This cohort study assessed allo-HCT at a single center from 2010 to 2021. Participants included all patients aged at least 18 years who underwent their first T-replete allo-HCT, with at least 6 months of follow-up. Data were analyzed from August 1 to December 15, 2022.

**Exposures:**

Antibiotics between 7 days before and 30 days after transplant.

**Main Outcomes and Measures:**

The primary outcome was grade II to IV aGVHD. The secondary outcome was grade III to IV aGVHD. Data were analyzed using 3 orthogonal methods: conventional Cox proportional hazard regression, marginal structural models, and machine learning.

**Results:**

A total of 2023 patients (median [range] age, 55 [18-78] years; 1153 [57%] male) were eligible. Weeks 1 and 2 after HCT were the highest-risk intervals, with multiple antibiotic exposures associated with higher rates of subsequent aGVHD. In particular, exposure to carbapenems during weeks 1 and 2 after allo-HCT was consistently associated with increased risk of aGVHD (minimum hazard ratio [HR] among models, 2.75; 95% CI, 1.77-4.28), as was week 1 after allo-HCT exposure to combinations of penicillins with a β-lactamase inhibitor (minimum HR among models, 6.55; 95% CI, 2.35-18.20).

**Conclusions and Relevance:**

In this cohort study of allo-HCT recipients, antibiotic choices and schedules in the early course of transplantation were associated with aGVHD rates. These findings should be considered in antibiotic stewardship programs.

## Key Points

**Question:**

Are antibiotics and antibiotic exposure timeframes associated with acute graft-vs-host disease (aGVHD) after allogeneic hematopoietic cell transplantation (allo-HCT)?

**Findings:**

In this cohort study including 2023 patients and using 3 orthogonal approaches applied to antibiotic use between 7 days before and 30 days after allo-HCT procedures, several antibiotics and exposure timeframes were found to be associated with aGVHD rates. Most notably, carbapenems and penicillins with a β-lactamase inhibitor used during the first 2 weeks after allo-HCT were consistently associated with increased hazard of aGVHD.

**Meaning:**

These findings suggest that aGVHD risk should be considered in antibiotic stewardship programs during allo-HCT.

## Teaser

This cohort study uses 3 modeling methods to identify antibiotics and antibiotic exposure timeframes associated with hazard of acute graft-vs-host disease after allogenic hematopoietic cell transplantation.

## Introduction

Antibiotics disrupt the gut microbiota in recipients of allogeneic hematopoietic cell transplantation (allo-HCT).^1,2^ Substantial evidence suggests that microbiota injury in the peritransplant period is associated with increased risk of acute graft-vs-host disease (aGVHD).^3,4,5,6,7,8,9,10,11,12^ Considering the extensive use of antibiotics in these patients and their powerful effects on the microbiota, a microbiota-cognizant use-and-choose wisely approach to antibiotic practice may thus reduce the risk of aGVHD. Currently, aGVHD risk is not considered in antibiotic stewardship programs.^13^

Several associations have been reported between antibiotics and aGVHD.^8,11,14,15,16,17,18,19,20,21^ Some antibiotics with potent antianaerobic activity (eg, carbapenems, piperacillin-tazobactam) have been associated with more aGVHD,^8,11,14,15,16,17,21^ although other antianaerobic antibiotics, such as metronidazole, have been associated with lower rates of aGVHD.^22^ Antibiotics are used in complex clinical situations; therefore, their effects may depend on when they are administered in relation to clinical events and whether other antibiotics are given before, during, or afterward. In such circumstances, the conventional Cox model may produce biased estimates. Marginal structural models overcome this limitation and improve causal inference.^23^ The multifactorial nature of aGVHD pathogenesis^24^ necessitates a large sample size and special statistical methods to elucidate independent associations for antibiotics. We conducted a single-center, retrospective study using a large database with daily antibiotic exposure data for more than 2000 allo-HCT procedures and an integrated statistical approach consisting of 3 orthogonal methods: proportional hazards regression, marginal structural model, and machine learning.

## Methods

This cohort study was approved by the Fred Hutchinson Cancer Center institutional review board. All patients provided written informed consent to participate in institutional database research. This study is reported following the Strengthening the Reporting of Observational Studies in Epidemiology (STROBE) reporting guideline.

We analyzed the allo-HCT database at the Fred Hutchinson Cancer Center with patients aged at least 18 years who underwent allo-HCT between 2010 and 2021, with at least 6 months of follow-up for survivors. Second allo-HCTs and ex vivo T-cell depleted allo-HCTs were not included. No other inclusion or exclusion criteria were used. Conditioning intensity and aGVHD staging and grading were defined according to the International Blood and Marrow Transplant Research criteria.^25,26^ Time to neutrophil engraftment was defined as the first of 3 consecutive days with absolute neutrophil count greater than 500/μL (to convert to  × 10^9^/L, multiply by 0.001).^27^ We considered oral and intravenous antibiotic exposures between 7 days before and 30 days after the procedure, with day 0 being the day of transplant. This interval was divided into 5 nonoverlapping intervals of approximately 1 week each. Antibiotics were classified into 17 classes: aminoglycosides, aztreonam, carbapenems, first- or second-generation cephalosporins, third- or higher-generation cephalosporins with or without a β lactamase inhibitor (2 separate groups), dalfopristin or quinupristin, fluoroquinolones, linezolid, macrolides, penicillins with or without a β lactamase inhibitor (2 separate groups), rifaximin, tetracyclines, trimethoprim-sulfamethoxazole, intravenous vancomycin, and oral vancomycin. Both inpatient and outpatient administrations were included. Patients received prophylactic antibiotics, usually fluoroquinolones, while neutropenic. Empirical treatment of neutropenic fever was generally third- or fourth-generation cephalosporins.

We evaluated 2 end points: grade II to IV (primary end point) and grade III to IV (secondary end point) aGVHD. We used 3 models to find factors associated with grade II to IV aGVHD. To ensure model stability in the analysis of grade III to IV aGVHD using models 1 and 2, we considered all nonantibiotic variables but only statistically significant antibiotic variables from the analysis of grade II to IV aGVHD. The grade III to IV aGVHD models did not converge when all antibiotic exposures were included. Therefore, the results for grade III to IV aGVHD should be treated with caution.

### Model 1: Proportional Hazards Regression With Time-Dependent Exposures

We fitted a multivariable proportional hazards model on the occurrence of aGVHD, in which death without GVHD was treated as a competing risk using the Fine and Gray model. The main variable was antibiotic exposure, coded as a binary categorical term per antibiotic class (each of the 9 most frequently used classes) and interval (each of the 5 intervals). Exposure to an antibiotic on different days of the same interval was assumed to have the same effect on GVHD hazard. Covariates in the model included factors most strongly associated with aGVHD in previous studies, including graft source, conditioning intensity, antithymocyte globulin use in conditioning, and an interaction term for donor type and GVHD prophylaxis. Two additional variables were included for neutrophil engraftment: a categorical variable coding whether neutrophil engraftment occurred or not and a continuous variable for days to neutrophil engraftment. For patients who did not engraft neutrophils, the median time to neutrophil engraftment was assigned to the latter variable. Thus, the regression coefficient for the categorical variable compares patients without neutrophil engraftment with a hypothetical patient experiencing neutrophil engraftment at median. We combined antibiotic use in adjacent intervals if there was perfect separation on event occurrence (eg, all or none of the patients exposed to an antibiotic in an interval developed aGVHD) to ensure model stability.

After fitting the full model, we conducted model selection using a backward elimination procedure in which in each step, antibiotic exposures with similar associations (significant and consistently positive or negative or not significant) in adjacent intervals were combined over those intervals and assumed to have a constant effect on GVHD hazard or antibiotic exposures with no significant association across all 5 intervals were removed. Models with smaller Akaike information criterion (AIC) values were preferred. Hazard ratios (HRs) and 95% CIs were calculated.

### Model 2: Marginal Structural Model

We used marginal structural models to estimate the association of time-dependent antibiotic exposures with aGVHD hazard in the presence of potentially confounding, previous, time-dependent antibiotic exposures. The model assumes that the vector of antibiotic exposures on a given day may depend on antibiotic exposures on the previous day and the baseline covariates, with antibiotic exposure probability estimated by a logistic regression model. The inverse-probability-of-treatment weighting approach with stabilized weight was used to estimate the model parameters (HRs and 95%CIs).^23^ The same intervals as in model 1 were used for antibiotic exposures, and death without aGVHD was considered a competing risk. The same procedures as in model 1 were used to derive the final version of model 2.

### Model 3: Machine Learning

We included 94 variables, including 85 antibiotic variables (17 types × 5 exposure intervals) as potential risk factors for aGVHD in the Boruta package,^28^ a random forest feature selection algorithm, in R statistical software version 4.2.0 (R Project for Statistical Computing). For each antibiotic, exposure in a given interval was coded as a binary variable (1 = exposed; 0 = not exposed). Similarly, grade II to IV aGVHD was coded as a binary variable, 1 if the patient developed the outcome by day 180 after allo-HCT and zero otherwise. Because Boruta is not suitable for time-to-event or competing risk analysis, patients who died without aGVHD before day 180 were excluded. The goal in the Boruta algorithm is to classify the potential risk factors into important and unimportant features. Possible multicollinearity among important features does not pose a problem in this algorithm because all such variables are selected as important.

Boruta operates in 5 steps. First, Boruta duplicates all features, shuffling the values of each added duplicate (“shadow” features), and merging original and shadow features into the same database. Second, Boruta trains a random forest classifier on the combined data set and determines the importance of each feature using 1 of the several available indices (here, mean decrease accuracy). Third, Boruta compares the score obtained for each original variable with the maximum score obtained from the shadow features. Original features with scores higher than this maximum receive a “hit” toward being important while those with scores lower than the maximum shadow score receive a “hit” toward being unimportant. Fourth, a set number of iterations (steps 1-3) are performed, assigning hits in each permutation. Finally, a *Z* score is calculated based on the hits with which it determines the features that performed significantly better than shadow (classified as important) and those that performed significantly worse than shadow (classified as unimportant).

**Figure 1.**
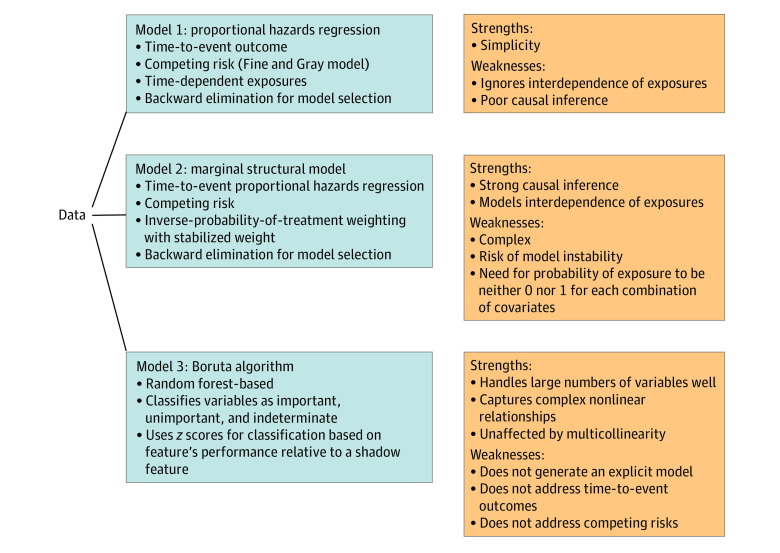
Summary of the 3 Statistical Methods Data were analyzed by these methods independently.

Figure 1 summarizes our statistical approach. While all methods seek to identify independent associations between time-dependent antibiotic exposures and aGVHD, each focuses on a different aspect of the question. No model by itself is perfect, but the 3 of them together reveal a more comprehensive perspective. Thus, consistency of the results across models is not an objective.

All analyses were performed in R version 4.2.0. *P* values were 2-sided, and statistical significance was set at *P* = .05. Data were analyzed from August 1 to December 15, 2022.

## Results

**Figure 2.**
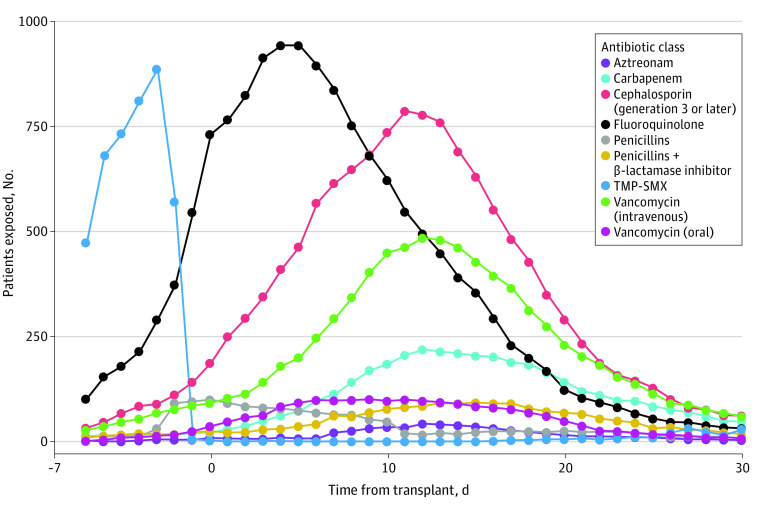
Antibiotic Exposures Between 7 Days and 30 Days After Allogeneic Hematopoietic Cell Transplantation Only the 9 most frequently used classes are shown for ease of visualization. TMP-SMX indicates trimethoprim-sulfamethoxazole.

Analyses included 2023 patients (median [range] age, 55 [18-78] years; 1153 [57%] male) (eTable 1 in Supplement 1). There were no missing data or loss to follow up. Antibiotic exposures are summarized in Figure 2. The most frequently used antibiotics were fluoroquinolones, third-generation or higher cephalosporins, intravenous vancomycin, trimethoprim-sulfamethoxazole (almost exclusively before the HCT procedure), and carbapenems. By day 180 after allo-HCT, 1461 patients (72%) developed grade II to IV aGVHD, with a median (IQR) time to onset of 29 (20-42) days after HCT. Our institutional rates of grade II to IV aGVHD have historically been higher than those reported by most other transplant centers, believed to be due to our low threshold for upper endoscopy, leading to more frequent diagnosis of upper gastrointestinal aGVHD. Indeed, grade III to IV aGVHD occurred in only 295 patients (15%). By 180 days after allo-HCT, 206 patients (14%) with grade II to IV aGVHD and 102 patients (35%) with grade III to IV aGVHD died. A total of 1990 patients (98%) engrafted neutrophils, at a median (IQR) of 17 (14-20) days after transplant.

### Model 1: Proportional Hazards Regression

#### Grade II to IV aGVHD

**Figure 3.**
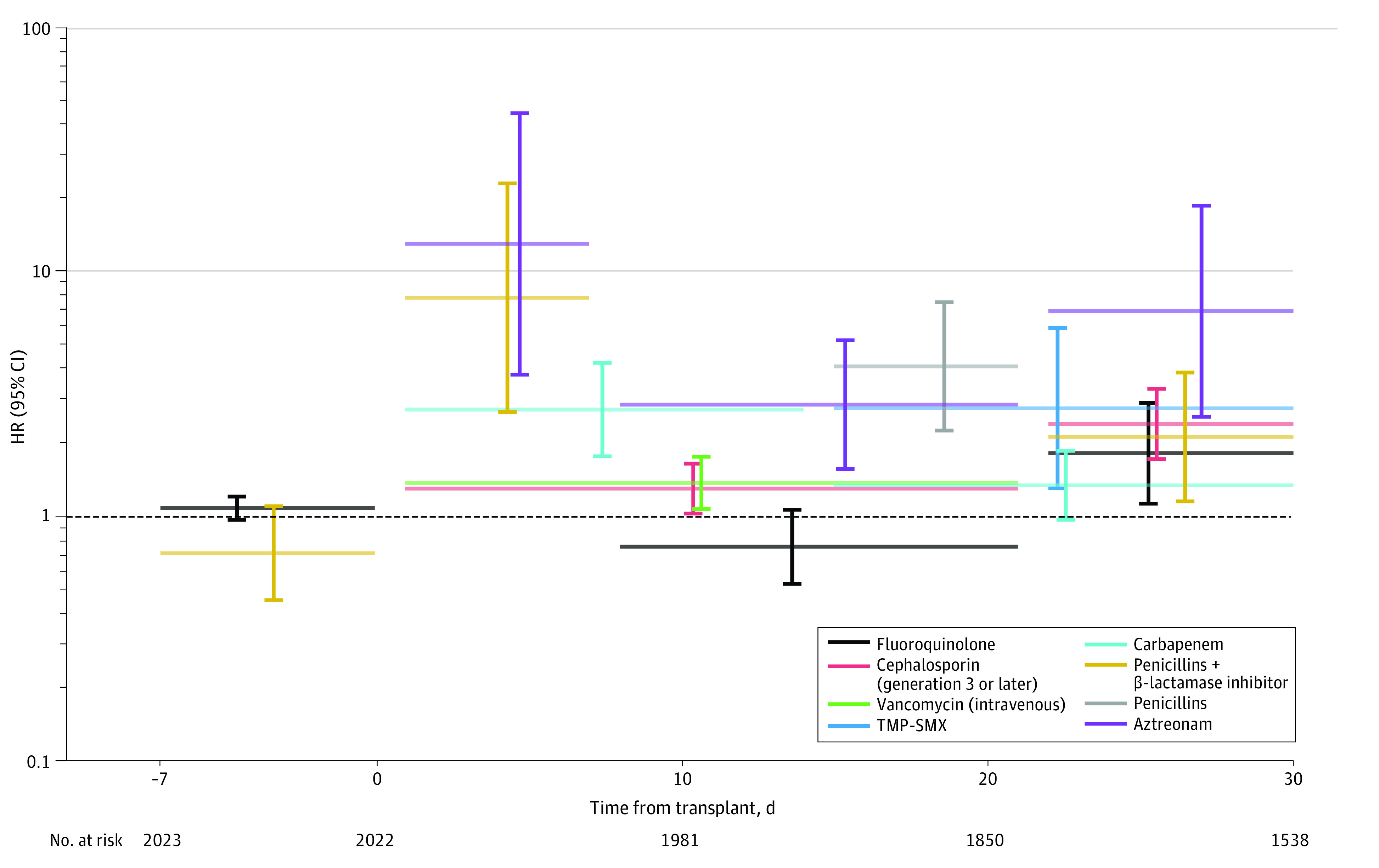
Parameter Estimates for Antibiotic Exposures in the Multivariable Proportional Hazards Model (Model 1) for Grade II to IV Acute Graft-vs-Host Disease Horizontal lines indicate the intervals for each exposure in the final version of the model. Hazard ratios (HRs) and their 95% CIs are plotted along vertical lines. CIs not crossing the dashed line (HR, 1.00) indicate statistically significant exposures (*P* < .05). TMP-SMX indicates trimethoprim-sulfamethoxazole.

The final version of model 1 for grade II to IV aGVHD (Figure 3; eTable 2 in Supplement 1) included all nonantibiotic covariates and antibiotic exposures. The variance inflation factor (VIF) across the included nonantibiotic covariates and antibiotic exposures in intervals ranged from 1.00 to 2.99, arguing against significant multicollinearity. The following exposures were associated with greater hazard of aGVHD: fluoroquinolones during week 4 after allo-HCT (HR, 1.82; 95% CI, 1.13-2.93), carbapenems during weeks 1 to 2 after allo-HCT (HR, 2.75; 95% CI, 1.77-4.28), penicillins with a β lactamase inhibitor during weeks 1 (HR, 7.90; 95% CI, 2.69-23.25) and 4 (HR, 2.13; 95% CI, 1.16-3.90) after allo-HCT, intravenous vancomycin during weeks 1 to 3 after allo-HCT (HR, 1.38; 95% CI, 1.07-1.76), third-generation or higher cephalosporins or aztreonam during weeks 1 to 4 after allo-HCT (weeks 1-3 cephalosporins: HR, 1.31; 95% CI, 1.03-1.65; week 4 cephalosporins: HR, 2.40; 95% CI, 1.72-3.35; week 1 aztreonam: HR, 13.14; 95% CI, 3.83-45.12; weeks 2-3 aztreonam: HR, 2.88; 95% CI, 1.57-5.29; week 4 aztreonam: HR, 6.96; 95% CI, 2.57-18.86), trimethoprim-sulfamethoxazole during weeks 3 to 4 after allo-HCT (HR, 2.78; 95% CI, 1.31-5.93), and penicillins during week 3 after allo-HCT (HR, 4.14; 95% CI, 2.26-7.58).

#### Grade III to IV aGVHD

The final version of model 1 for grade III to IV aGVHD is shown in eTable 3 and eFigure 1 in Supplement 1. The following exposures were associated with greater hazard of aGVHD: fluoroquinolones during the week before HCT (HR, 1.45; 95% CI, 1.14-1.85) and week 4 after allo-HCT (HR, 2.70; 95% CI, 1.30-5.59), penicillins with a β lactamase inhibitor during weeks 1 (HR, 14.16; 95% CI, 2.71-74.07) and 4 (HR, 4.14; 95% CI, 1.76-9.77) after allo-HCT, intravenous vancomycin or aztreonam during weeks 1 to 3 after allo-HCT (vancomycin: HR, 1.7; 95% CI, 1.04-2.76; week 1 aztreonam: HR, 50.56; 95% CI, 11.46-223.06; weeks 2-3 aztreonam: HR, 4.38; 95% CI, 1.64-11.69), third-generation or higher cephalosporins during week 4 after allo-HCT (HR, 2.73; 95% CI, 1.49-5.01), and trimethoprim-sulfamethoxazole during weeks 3 to 4 after allo-HCT (HR, 7.82; 95% CI, 3.05-20.03).

### Model 2: Marginal Structural Model

#### Grade II to IV aGVHD

**Figure 4.**
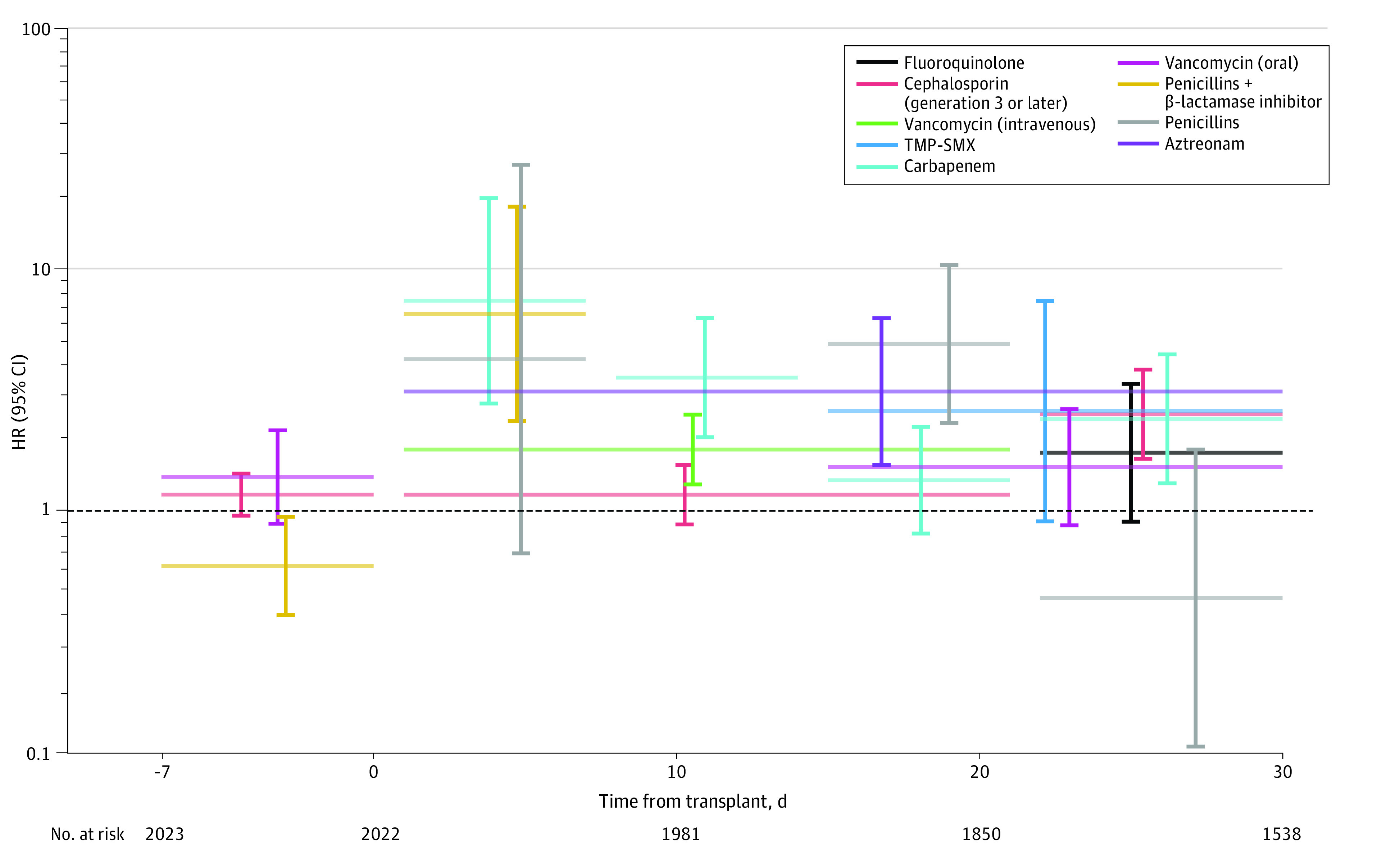
Parameter Estimates for Antibiotic Exposures in the Marginal Structural Model (Model 2) for Grade II to IV Acute Graft-vs-Host Disease The horizontal lines indicate the intervals for each exposure in the final version of the model. Hazard ratios (HRs) and their 95% CIs are plotted along vertical lines. CIs not crossing the dashed line (HR, 1.00) indicate statistically significant exposures (*P* < .05). TMP-SMX indicates trimethoprim-sulfamethoxazole.

The final version of the marginal structural model 2 of grade II to IV aGVHD (Figure 4; eTable 4 in Supplement 1) included all nonantibiotic covariates and antibiotic exposures. VIF values across the included nonantibiotic covariates and antibiotic exposures in intervals ranged from 1.03 to 3.15, arguing against significant multicollinearity. The overall pattern of findings was similar to model 1. An association was found for carbapenems during weeks 1 (HR, 7.42; 95% CI, 2.78-19.76), 2 (HR, 3.56; 95% CI, 2.02-6.29), and 4 (HR, 2.40; 95% CI, 1.30-4.45) after allo-HCT. Exposure to penicillins with a β lactamase inhibitor during week 1 after allo-HCT was associated with greater hazard of aGVHD (HR, 6.55; 95% CI, 2.35-18.20), while exposure to these antibiotics before HCT was associated with lower hazard of aGVHD (HR, 0.59; 95% CI, 0.37-0.94).

#### Grade III to IV aGVHD

The final version of model 2 for grade III to IV aGVHD is shown in eTable 5 and eFigure 2 in Supplement 1. The following antibiotic exposures were associated with greater hazard of aGVHD: penicillins with a β lactamase inhibitor during week 1 after allo-HCT (HR, 7.82; 95% CI, 1.70-36.09), carbapenems during weeks 2 (HR, 3.31; 95% CI, 1.17-9.39) and 4 (HR, 4.44; 95% CI, 1.93-10.19) after allo-HCT, intravenous vancomycin during weeks 1 to 3 after allo-HCT (HR, 2.26; 95% CI, 1.27-4.05), oral vancomycin before HCT (HR, 2.99; 95% CI, 1.59-5.65) and during weeks 3 to 4 after allo-HCT (HR, 2.78; 95% CI, 1.23-6.26), aztreonam during weeks 1 to 4 after allo-HCT (HR, 4.10; 95% CI, 1.70-9.87), and trimethoprim-sulfamethoxazole during weeks 3 to 4 after allo-HCT (HR, 10.78; 95% CI, 3.63-32.01).

### Model 3: Machine Learning

**Figure 5.**
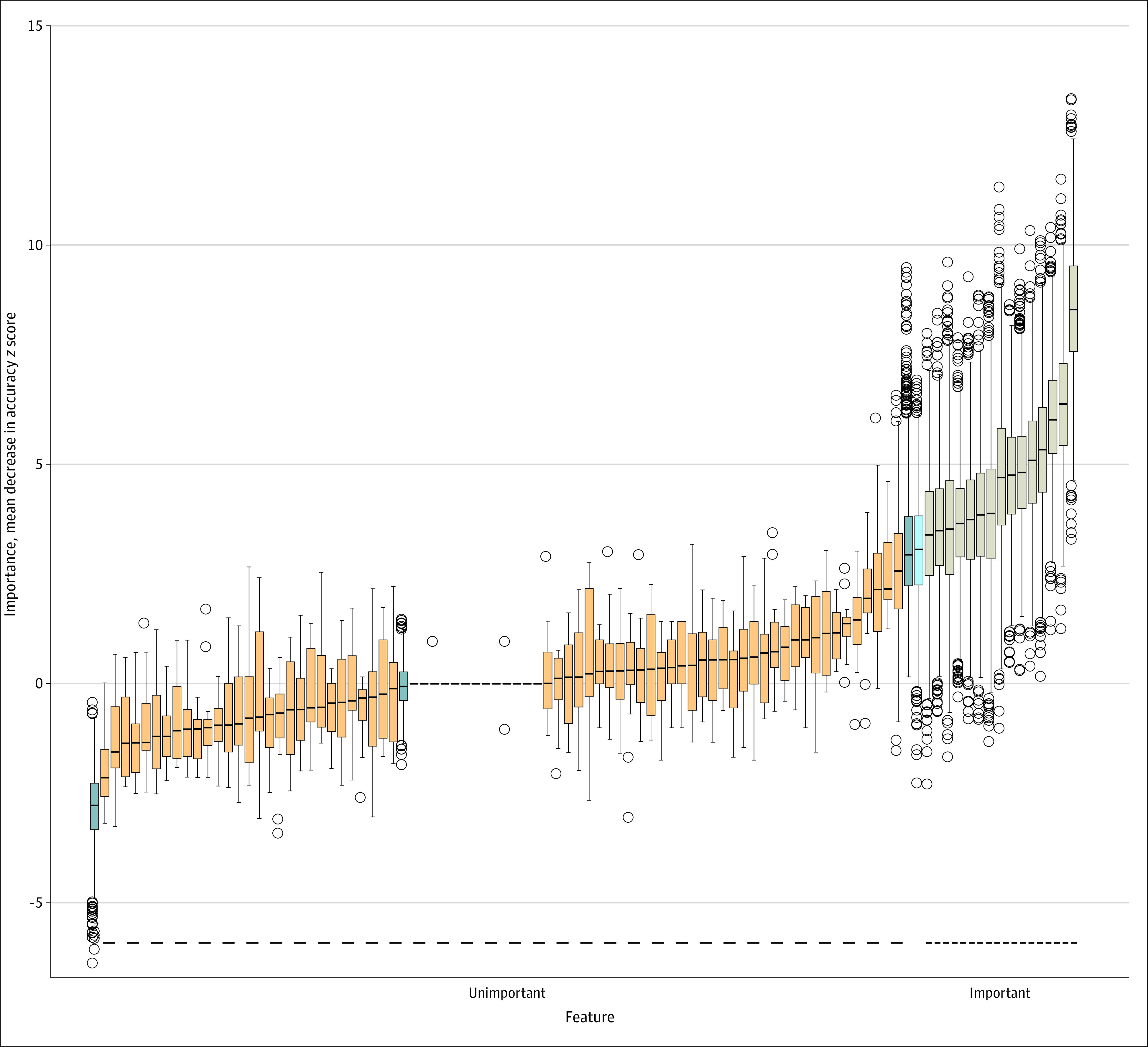
Results of Boruta Random Forest Features are sorted according to their importance value measured as mean decrease accuracy. Tan bars indicate important features; orange, unimportant features; light blue, indeterminate features; and dark blue, shadow features. Features are listed in the eTable in Supplement 2.

Using 2000 iterations, Boruta applied to grade II to IV aGVHD was able to classify all features except 1. Fifteen features were classified as important (eTable in Supplement 2; Figure 5), with the top 3 being conditioning intensity, donor type, and carbapenem exposure during week 1 after allo-HCT. The only variable that could not be classified with certainty was pre-HCT exposure to penicillins with a β lactamase inhibitor. An additional 500 iterations placed this variable among important variables. The grade III to IV aGVHD model could not be built due to its instability in classification of different antibiotics.

## Discussion

In this cohort study, we performed a comprehensive analysis of antibiotic exposures as a risk factor for aGVHD after allo-HCT. We included 94 variables (17 antibiotic groups over 5 week-long intervals and 9 nonantibiotic variables covariates) in 3 orthogonal analytical approaches using a database of more than 2000 allo-HCT procedures performed over a decade. The most consistent finding was the association for carbapenem exposure during week 1 (and to a lesser extent week 2) after allo-HCT. In murine studies, carbapenem led to the expansion of obligate anaerobic, mucus-degrading species, such as *Akkermansia muciniphila*^8^ and *Bacteroides thetaiotaomicron*,^11^ resulting in impaired gut barrier integrity and more aGVHD. The association between carbapenem exposure and aGVHD was also observed in a large previous study (approximately 1200 patients; HR, 1.3) and several smaller cohorts.^15,17^ In another large series (approximately 1200 patients), exposure to carbapenems or piperacillin-tazobactam (grouped together as antianaerobic antibiotics) in the pre-engraftment period was associated with greater risk of aGVHD (HR, 1.3).^16^ By distinguishing different intervals and considering prior antibiotic exposures as time-dependent covariates, we found an association for carbapenems during weeks 1 and 2 after allo-HCT, with HRs ranging from 2.75 to 7.42.

Another antibiotic class with a significant association during week 1 after allo-HCT was penicillins with a β lactamase inhibitor, with HRs ranging from 6.55 to 7.90. The most frequently used antibiotic in this class is piperacillin-tazobactam, found in some previous reports to be associated with greater risk of aGVHD or mortality from aGVHD.^8,14,16^ Notably, exposure to any of the 9 most frequently used antibiotics in at least 1 of the 5 intervals was associated with greater hazard of aGVHD. This finding suggests that while specific bacteria may influence the risk of aGVHD, there are also more general patterns of microbiota injury that may be important in aGVHD pathogenesis. This hypothesis is supported by a 2012 study^9^ showing that large microbial fluctuations in the peritransplant interval may increase aGVHD risk. Weeks 1 and 2 after allo-HCT appeared to be the highest-risk intervals, with multiple antibiotic exposure associated with greater hazard of aGVHD. These intervals represent the pre-engraftment period, when the allogeneic graft is rapidly expanding and its immune effector cells are coming into contact with the changing gut microbiota. Studies have shown that microbiota changes start to occur within days after antibiotic exposure.^29,30^

An unexpected finding was the association between pre-HCT exposure to penicillins with a β lactamase inhibitor and lower rates of both grade II to IV and III to IV aGVHD across all 3 methods. Although a statistically significant result was obtained only for grade II to IV aGVHD (model 2), the remarkably consistent pattern and similar HRs across models and for both end points may suggest a true biological relationship. The potential value of early broad-spectrum suppression of the gut microbiota and its possible protective effect against aGVHD has been debated for years. The best evidence for such an effect comes from a 1999 randomized trial by Beelen et al^22^ of ciprofloxacin plus metronidazole (experimental group) vs ciprofloxacin (control group) during the first 5 weeks after HCT. A reduction of grade II aGVHD incidence by 50% was observed in the experimental group, and this was associated with a reduction of anaerobic bacteria in the stool (culture-based analysis).^22^ In addition, patients who developed grade II to IV aGVHD had a significantly higher anaerobic bacteria load in the stool.^22^ A few other gut decontamination studies observed similar results, including some that started this strategy before HCT.^31,32^ Most of these studies were performed in the premicrobiota era, thus the precise effect of the intervention on the microbiota and its possible involvement in the causal link cannot be ascertained.

In addition to mechanistic murine studies, there are many questions open to further investigation. First, are our findings consistent among different centers? Second, would posttransplant replacement of strong antianaerobic antibiotics with anaerobe-sparing antibiotics lead to less aGVHD? This could be tested in a randomized trial, and one such trial is currently ongoing (ClinicalTrials.gov identifier NCT03078010). Third, considering the negative association between pre-HCT use of penicillins with a β lactamase inhibitor and aGVHD, would pre-HCT use of this antibiotic class instead of fluoroquinolones reduce risk of aGVHD? Fourth, are patients with penicillin or cephalosporin allergies more likely to develop aGVHD (since they are more likely to receive carbapenems or piperacillin-tazobactam after HCT)? Fifth, could antibiotic exposures be used to identify patients at higher risk for aGVHD and direct them to microbiota therapeutic trials?

### Limitations

This study has some limitations. The reason for choosing a specific type of antibiotic among several possible choices cannot always be ascertained in retrospective studies, indicating the possibility of unmeasured confounders. Antibiotic use patterns partially depend on evolving local epidemiological patterns for common multidrug-resistant organisms. The rates of vancomycin-resistant *Enterococcus* and methicillin-resistant *Staphylococcus aureus* infections in our patients were low during this study.^33,34^ Our *Clostridioides difficile* infection rates have been approximately 10% in the last several years.^35^ We see 1 to 3 infections with carbapenem-resistant *Enterobacterales* per year. Extended-spectrum β-lactamase colonization rates among our patients is approximately 3%.

## Conclusions

The findings of this cohort study suggest that several antibiotics commonly used to treat neutropenic fever and infections after HCT were associated with increased rates of aGVHD. The most consistent finding in this study and prior reports, and with recent mechanistic support from murine studies, is a detrimental association of aGVHD with carbapenem exposure in weeks 1 and 2 after HCT. Avoiding this class of antibiotics early after transplant seems prudent. The challenge in current practice is the lack of antibiotics that are effective enough in prophylaxis or treatment but spare the commensal microbiota.^18^ Various microbiota-targeted approaches are being tested to protect the microbiota during antibiotic use or restore it after injury.^36^ Examples of potentially protective treatments are nonselective luminal adsorbents, such as oral activated charcoal^37^ and selective luminal antibiotic degraders, such as β-lactamases^38^ and metallo-β-lactamases.^39^ Examples of potentially restorative treatments include prebiotics^40,41^ and fecal microbiota transplantation.^42,43,44,45^ If our results are replicated in independent cohorts, antibiotic-associated risk of aGVHD could become a consideration in antibiotic stewardship programs.

## Notes

Supplement 1. **eTable 1. **Patient Characteristics **eTable 2.** Parameter Estimates for Nonantibiotic Variables in the Multivariable Proportional Hazards Model for Grade II-IV Acute GVHD, Where the Effect of Each Antibiotic Is Constant in Each Interval **eTable 3.** Parameter Estimates for Nonantibiotic Variables in the Multivariable Proportional Hazards Model for Grade III-IV Acute GVHD, Where the Effect of Each Antibiotic Is Constant in Each Interval **eTable 4.** Parameter Estimates for Nonantibiotic Variables in the Marginal Structural Model for Grade II-IV Acute GVHD, Where the Effect of Each Antibiotic Is Constant in Each Interval **eTable 5.** Parameter Estimates for Nonantibiotic Variables in the Marginal Structural Model for Grade III-IV Acute GVHD, Where the Effect of Each Antibiotic Is Constant in Each Interval **eFigure 1.** Parameter Estimates for Antibiotic Exposures in the Multivariable Proportional Hazards Model (Model 1) for Grade III-IV Acute GVHD **eFigure 2.** Parameter Estimates for Antibiotic Exposures in the Marginal Structural Model (Model 2) for Grade III-IV Acute GVHD

Supplement 2. **eTable.** Boruta Output

Supplement 3.
Data Sharing Statement
