# Supplementary material for: Analysis of Antibiotic Exposure and Development of Acute Graft-vs-Host Disease Following Allogeneic Hematopoietic Cell Transplantation
Source: JAMA Netw Open. 2023 Jun 7;6(6):e2317188. doi: 10.1001/jamanetworkopen.2023.17188 (PMC10248746; doi:10.1001/jamanetworkopen.2023.17188)
Supplement: Supplement 1. — eTable 1. Patient Characteristics eTable 2. Parameter Estimates for Nonantibiotic Variables in the Multivariable Proportional Hazards Model for Grade II-IV Acute GVHD, Where the Effect of Each Antibiotic Is Constant in Each Interval eTable 3. Parameter Estimates for Nonantibiotic Variables in the Multivariable Proportional Hazards Model for Grade III-IV Acute GVHD, Where the Effect of Each Antibiotic Is Constant in Each Interval eTable 4. Parameter Estimates for Nonantibiotic Variables in the Marginal Structural Model for Grade II-IV Acute GVHD, Where the Effect of Each Antibiotic Is Constant in Each Interval eTable 5. Parameter Estimates for Nonantibiotic Variables in the Marginal Structural Model for Grade III-IV Acute GVHD, Where the Effect of Each Antibiotic Is Constant in Each Interval eFigure 1. Parameter Estimates for Antibiotic Exposures in the Multivariable Proportional Hazards Model (Model 1) for Grade III-IV Acute GVHD eFigure 2. Parameter Estimates for Antibiotic Exposures in the Marginal Structural Model (Model 2) for Grade III-IV Acute GVHD [file jamanetwopen-e2317188-s001.pdf]

Supplemental Online Content

Rashidi A, Gao F, Fredricks DN, et al. Analysis of antibiotic exposure and development of acute graft-vs-host disease following allogeneic hematopoietic cell transplantation. *JAMA Netw Open*. 2023;6(6):e2317188. doi:10.1001/jamanetworkopen.2023.17188

eTable 1. Patient Characteristics

eTable 2. Parameter Estimates for Nonantibiotic Variables in the Multivariable Proportional Hazards Model for Grade II-IV Acute GVHD, Where the Effect of Each Antibiotic Is Constant in Each Interval

eTable 3. Parameter Estimates for Nonantibiotic Variables in the Multivariable Proportional Hazards Model for Grade III-IV Acute GVHD, Where the Effect of Each Antibiotic Is Constant in Each Interval

eTable 4. Parameter Estimates for Nonantibiotic Variables in the Marginal Structural Model for Grade II-IV Acute GVHD, Where the Effect of Each Antibiotic Is Constant in Each Interval

eTable 5. Parameter Estimates for Nonantibiotic Variables in the Marginal Structural Model for Grade III-IV Acute GVHD, Where the Effect of Each Antibiotic Is Constant in Each Interval

eFigure 1. Parameter Estimates for Antibiotic Exposures in the Multivariable Proportional Hazards Model (Model 1) for Grade III-IV Acute GVHD

eFigure 2. Parameter Estimates for Antibiotic Exposures in the Marginal Structural Model (Model 2) for Grade III-IV Acute GVHD

This supplemental material has been provided by the authors to give readers additional information about their work.

© 2023 Rashidi A et al. *JAMA Network Open.*

eTable 1. Patient Characteristics

N	2,023
Age, median (range)	55 (18-78) years
Sex, male (%)	1,153 (57%)
Donor type, n (%)	
HLA-matched unrelated	1,011 (50%)
HLA-matched sibling	463 (23%)
Cord blood	225 (11%)
HLA-mismatched unrelated	176 (9%)
HLA haploidentical	148 (7%)
Graft source, n (%)	
Peripheral blood	1,619 (80%)
Cord blood	225 (11%)
Bone marrow	179 (9%)
Conditioning intensity, n (%)	
Reduced intensity	907 (45%)
Myeloablative with high-dose TBI	368 (18%)
Myeloablative without high-dose TBI	748 (37%)
GVHD prophylaxis	
Calcineurin inhibitor-based	951 (47%)
Methotrexate-based	755 (37%)
PTCy-based	317 (16%)
ATG in conditioning, n (%)	66 (3%)
Days to neutrophil engraftment, median (range)	17 (2-64)

ATG: anti-thymocyte globulin, GVHD: graft-versus-host disease, HLA: Human leukocyte antigen, PTCy: post-transplantation cyclophosphamide, TBI: total body irradiation

© 2023 Rashidi A et al. *JAMA Network Open.*

eTable 2. Parameter Estimates for Nonantibiotic Variables in the Multivariable Proportional Hazards Model for Grade II-IV Acute GVHD, Where the Effect of Each Antibiotic Is Constant in Each Interval

Covariate	HR	95%CI	p
HLA haploidentical, non-PTCy-based prophylaxis[1]	1.12	(0.64, 1.97)	0.68
HLA haploidentical, PTCy-based prophylaxis[1]	0.91	(0.69, 1.18)	0.46
HLA-mismatched unrelated, non-PTCy-based prophylaxis[1]	0.94	(0.74, 1.2)	0.61
HLA-mismatched unrelated, PTCy-based prophylaxis[1]	0.84	(0.45, 1.56)	0.58
HLA-matched sibling, non-PTCy-based prophylaxis[1]	**0.69**	**(0.56, 0.84)**	**<0.001**
HLA-matched sibling, PTCy-based prophylaxis[1]	0.68	(0.45, 1.04)	0.08
HLA-matched unrelated, non-PTCy-based prophylaxis[1]	0.86	(0.72, 1.03)	0.11
HLA-matched unrelated, PTCy-based prophylaxis[1]	1.00	(0.77, 1.3)	1.00
Graft source: bone marrow vs. peripheral blood	1.03	(0.83, 1.28)	0.77
MAC without vs. with high-dose TBI	0.86	(0.75, 1.002)	0.05
RIC vs. MAC with high-dose TBI	**0.71**	**(0.61, 0.83)**	**$<10^{-4}$**
ATG use in conditioning (yes vs. no)	0.74	(0.51, 1.07)	0.11
Neutrophil engraftment (yes vs. no)	**10.30**	**(3.85, 27.54)**	**$<10^{-5}$**
Time to neutrophil engraftment	**0.97**	**(0.96, 0.98)**	**$<10^{-6}$**

[1]All compared to cord blood, non-PTCy-based prophylaxis. Values for significant variables are shown in bold. 95%CI: 95% confidence interval for hazard ratio, ATG: anti-thymocyte globulin, HLA: Human leukocyte antigen, HR: hazard ratio, MAC: myeloablative conditioning, MSD: matched sibling donor, MMUD: mismatched unrelated donor, MUD: matched unrelated donor, PTCy: post-transplantation cyclophosphamide, RIC: reduced-intensity conditioning, TBI: total body irradiation

eTable 3. Parameter Estimates for Nonantibiotic Variables in the Multivariable Proportional Hazards Model for Grade III-IV Acute GVHD, Where the Effect of Each Antibiotic Is Constant in Each Interval

Covariate	HR	95%CI	p
HLA haploidentical, non-PTCy-based prophylaxis[1]	0.45	(0.06, 3.42)	0.44
HLA haploidentical, PTCy-based prophylaxis[1]	0.69	(0.36, 1.31)	0.26
HLA-mismatched unrelated, non-PTCy-based prophylaxis[1]	1.33	(0.82, 2.17)	0.25
HLA-mismatched unrelated, PTCy-based prophylaxis[1]	0.86	(0.20, 3.64)	0.84
HLA-matched sibling, non-PTCy-based prophylaxis[1]	**0.54**	**(0.34, 0.86)**	**0.009**
HLA-matched sibling, PTCy-based prophylaxis[1]	0.52	(0.18, 1.51)	0.23
HLA-matched unrelated, non-PTCy-based prophylaxis[1]	1.00	(0.68, 1.47)	1.00
HLA-matched unrelated, PTCy-based prophylaxis[1]	0.99	(0.56, 1.76)	0.97
Graft source: bone marrow vs. peripheral blood	1.23	(0.78, 1.94)	0.36
MAC without vs. with high-dose TBI	0.90	(0.64, 1.25)	0.52
RIC vs. MAC with high-dose TBI	0.86	(0.61, 1.21)	0.38
ATG use in conditioning (yes vs. no)	0.61	(0.26, 1.43)	0.26
Neutrophil engraftment (yes vs. no)	4.02	(0.99, 16.23)	0.05
Time to neutrophil engraftment	0.98	(0.96, 1.005)	0.12

[1]All compared to cord blood, non-PTCy-based prophylaxis. Values for significant variables are shown in bold. 95%CI: 95% confidence interval for hazard ratio, ATG: anti-thymocyte globulin, HLA: Human leukocyte antigen, HR: hazard ratio, MAC: myeloablative conditioning, MSD: matched sibling donor, MMUD: mismatched unrelated donor, MUD: matched unrelated donor, PTCy: post-transplantation cyclophosphamide, RIC: reduced-intensity conditioning, TBI: total body irradiation

© 2023 Rashidi A et al. *JAMA Network Open.*

eTable 4. Parameter Estimates for Nonantibiotic Variables in the Marginal Structural Model for Grade II-IV Acute GVHD, Where the Effect of Each Antibiotic Is Constant in Each Interval

Covariate	HR	95%CI	p
HLA-haploidentical, non-PTCy-based prophylaxis[1]	1.31	(0.57, 3.02)	0.53
HLA-haploidentical, PTCy-based prophylaxis[1]	**0.74**	(0.56, 0.99)	**0.04**
HLA-mismatched unrelated, non-PTCy-based prophylaxis[1]	0.91	(0.71, 1.18)	0.49
HLA-mismatched unrelated, PTCy-based prophylaxis[1]	0.82	(0.37, 1.82)	0.62
HLA-matched sibling, non-PTCy-based prophylaxis[1]	**0.65**	(0.53, 0.80)	**$<10^{-4}$**
HLA-matched sibling, PTCy-based prophylaxis[1]	**0.64**	(0.42, 0.98)	**0.04**
HLA-matched unrelated, non-PTCy-based prophylaxis[1]	0.83	(0.68, 1.00)	0.05
HLA-matched unrelated, PTCy-based prophylaxis[1]	0.90	(0.67, 1.20)	0.47
Graft source: bone marrow vs. peripheral blood	0.99	(0.77, 1.28)	0.97
MAC without vs. with high-dose TBI	0.89	(0.76, 1.04)	0.15
RIC vs. MAC with high-dose TBI	**0.76**	(0.64, 0.89)	**$<10^{-3}$**
ATG use in conditioning (yes vs. no)	0.75	(0.41, 1.37)	0.34
Neutrophil engraftment (yes vs. no)	**10.77**	(3.71, 31.22)	**$<10^{-4}$**
Time to neutrophil engraftment	**0.97**	(0.96, 0.98)	**$<10^{-6}$**

[1]All compared to cord blood, non-PTCy-based prophylaxis. Values for significant variables are shown in bold. 95%CI: 95% confidence interval for hazard ratio, ATG: anti-thymocyte globulin, HLA: Human leukocyte antigen, HR: hazard ratio, MAC: myeloablative conditioning, MSD: matched sibling donor, MMUD: mismatched unrelated donor, MUD: matched unrelated donor, PTCy: post-transplantation cyclophosphamide, RIC: reduced-intensity conditioning, TBI: total body irradiation

eTable 5. Parameter Estimates for Nonantibiotic Variables in the Marginal Structural Model for Grade III-IV Acute GVHD, Where the Effect of Each Antibiotic Is Constant in Each Interval

Covariate	HR	95%CI	p
HLA-haploidentical, non-PTCy-based prophylaxis[1]	0.36	(0.05, 2.51)	0.30
HLA-haploidentical, PTCy-based prophylaxis[1]	**0.37**	**(0.18, 0.75)**	**0.006**
HLA-mismatched unrelated, non-PTCy-based prophylaxis[1]	1.07	(0.63, 1.81)	0.80
HLA-mismatched unrelated, PTCy-based prophylaxis[1]	0.68	(0.16, 2.86)	0.59
HLA-matched sibling, non-PTCy-based prophylaxis[1]	**0.43**	**(0.26, 0.71)**	**<0.001**
HLA-matched sibling, PTCy-based prophylaxis[1]	**0.27**	**(0.09, 0.86)**	**0.03**
HLA-matched unrelated, non-PTCy-based prophylaxis[1]	0.82	(0.55, 1.23)	0.33
HLA-matched unrelated, PTCy-based prophylaxis[1]	0.73	(0.37, 1.41)	0.35
Graft source: bone marrow vs. peripheral blood	1.26	(0.78, 2.03)	0.35
MAC without vs. with high-dose TBI	0.87	(0.6, 1.25)	0.45
RIC vs. MAC with high-dose TBI	0.92	(0.63, 1.34)	0.67
ATG use in conditioning (yes vs. no)	0.54	(0.23, 1.25)	0.15
Neutrophil engraftment (yes vs. no)	3.78	(0.87, 16.5)	0.08
Time to neutrophil engraftment	0.98	(0.95, 1.02)	0.31

[1]All compared to cord blood, non-PTCy-based prophylaxis. Values for significant variables are shown in bold. 95%CI: 95% confidence interval for hazard ratio, ATG: anti-thymocyte globulin, HLA: Human leukocyte antigen, HR: hazard ratio, MAC: myeloablative conditioning, MSD: matched sibling donor, MMUD: mismatched unrelated donor, MUD: matched unrelated donor, PTCy: post-transplantation cyclophosphamide, RIC: reduced-intensity conditioning, TBI: total body irradiation

© 2023 Rashidi A et al. *JAMA Network Open.*

eFigure 1. Parameter Estimates for Antibiotic Exposures in the Multivariable Proportional Hazards Model (Model 1) for Grade III-IV Acute GVHD

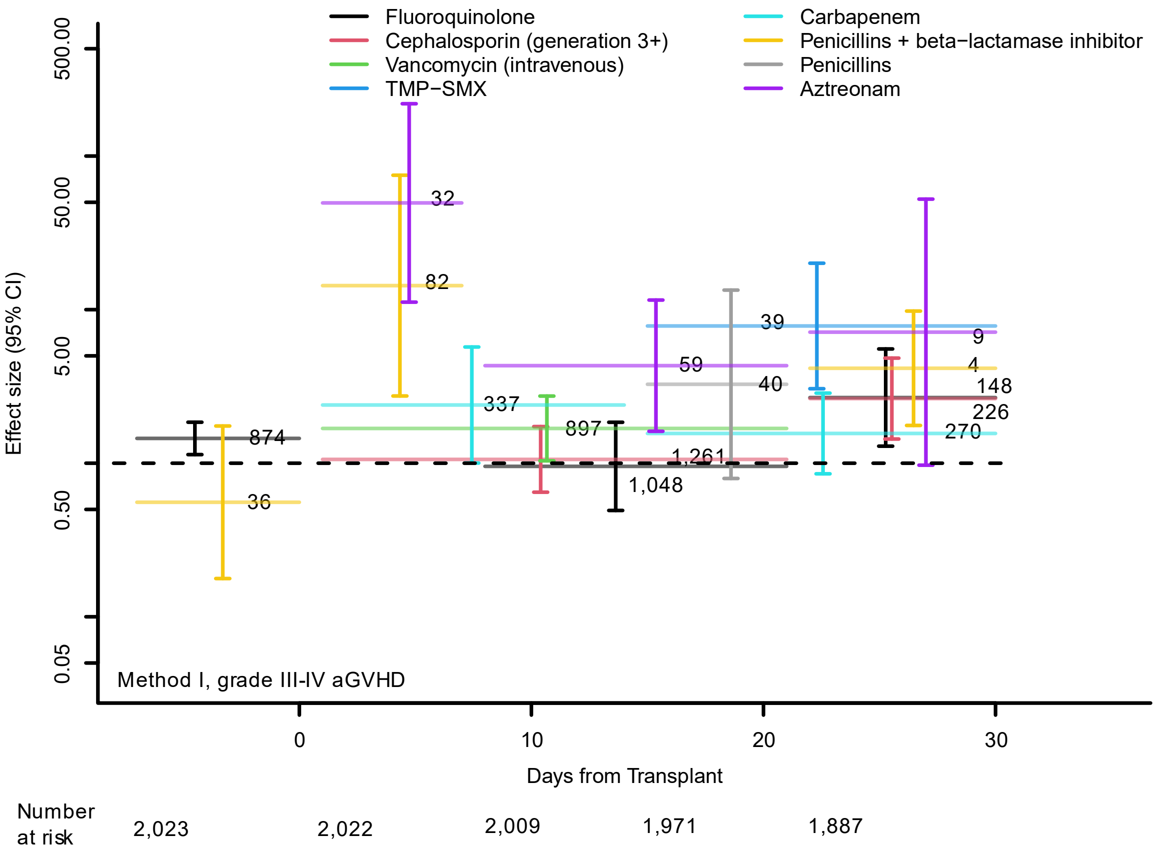

The horizontal lines indicate the intervals for each exposure in the final model (see Methods for full details). Numbers at the bottom of the plot show total at risk in the beginning of each interval. The number above each interval within the plot indicates the number of patients at risk (i.e., alive and without grade III-IV aGVHD) who became exposed to the corresponding antibiotic during that interval. Hazard ratios and their 95% confidence intervals are plotted along vertical lines. Confidence intervals not crossing the dashed line (hazard ratio 1) indicate statistically significant exposures ($p < 0.05$). TMP-SMX: trimethoprim-sulfamethoxazole, aGVHD: acute graft-versus-host disease

© 2023 Rashidi A et al. *JAMA Network Open.*

eFigure 2. Parameter Estimates for Antibiotic Exposures in the Marginal Structural Model (Model 2) for Grade III-IV Acute GVHD

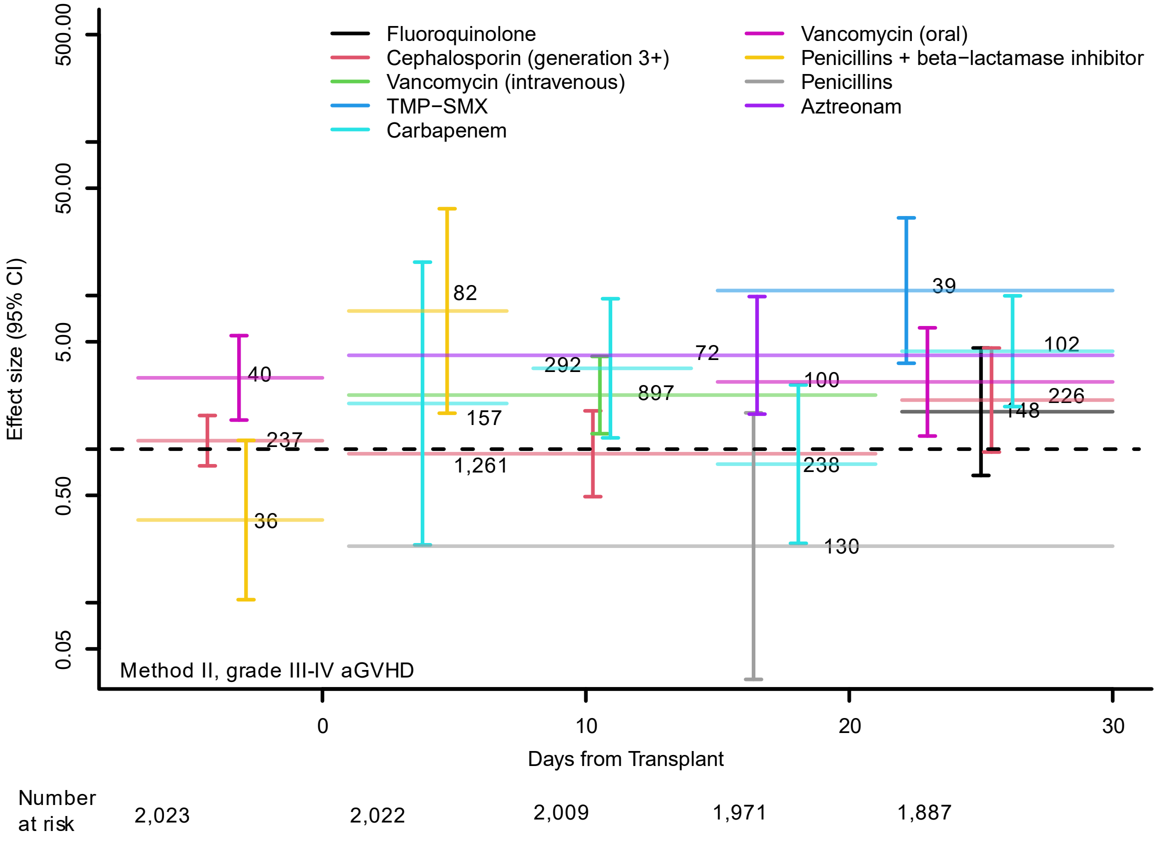

The horizontal lines indicate the intervals for each exposure in the final model (see Methods for full details). Numbers at the bottom of the plot show total at risk in the beginning of each interval. The number above each interval within the plot indicates the number of patients at risk (i.e., alive and without grade III-IV aGVHD) who became exposed to the corresponding antibiotic during that interval. Hazard ratios and their 95% confidence intervals are plotted along vertical lines. Confidence intervals not crossing the dashed line (hazard ratio 1) indicate statistically significant exposures ($p < 0.05$). TMP-SMX: trimethoprim-sulfamethoxazole, aGVHD: acute graft-versus-host disease

© 2023 Rashidi A et al. *JAMA Network Open.*
