# Supplementary material for: Analysis of Antibiotic Exposure and Development of Acute Graft-vs-Host Disease Following Allogeneic Hematopoietic Cell Transplantation
Source: JAMA Netw Open. 2023 Jun 7;6(6):e2317188. doi: 10.1001/jamanetworkopen.2023.17188 (PMC10248746; doi:10.1001/jamanetworkopen.2023.17188)
Supplement: Supplement 3. — Data Sharing Statement [file jamanetwopen-e2317188-s003.pdf]

Data Sharing Statement

Rashidi. Analysis of Antibiotic Exposure and Development of Acute Graft-vs-Host Disease Following Allogeneic Hematopoietic Cell Transplantation. *JAMA Netw Open*. Published June 07, 2023. doi:10.1001/jamanetworkopen.2023.17188

Data
Data available: No
